# Lysolecithin Improves Broiler Growth Performance through Upregulating Growth-Related Genes and Nutrient Transporter Genes Expression Independent of Experimental Diet Nutrition Level

**DOI:** 10.3390/ani12233365

**Published:** 2022-11-30

**Authors:** Zhiming Zhang, Song Zhang, Kangkang Nie, He Zheng, Zheng Luo, In-Ho Kim

**Affiliations:** 1 Kemin (China) Technologies Co., Ltd., 25 Qinshi Road, Sanzao, Zhuhai 519040, China; 2 Department of Animal Resource & Science, Dankook University, Cheonan 330-714, Republic of Korea

**Keywords:** Lysolecithin, growth performance, transporter genes, growth-related genes, nutrition

## Abstract

**Simple Summary:**

Lysolecithin derived from hydrolysis of soy lecithin has been widely used as emulsifier in the feed industry to improve growth performance of farm animals for some time. The components of Lysolecithin have proved to have multiple functions, but the real mechanism of action of Lysolecithin on growth promotion in the animal is still unclear. To investigate how it works, we executed this trial. The results show that, besides improving growth performance of broilers, supplementation of Lysolecithin also improved ileal digestibility of amino acids. Further studies found that nutrient (amino acids and fatty acids) transporter genes in the small intestine and growth-related genes in the liver and muscle of broilers were significantly upregulated by supplementation of Lysolecithin independent of experimental diet nutrition level. Upregulating the nutrient transporter gene and growth-related gene expression of the host might be the action mechanism of lysolecithin on growth promotion in animals. By understanding its mechanism of action, researchers can develop new feed additives with better function and lower cost to promote the growth of farm animals and meet the ever-growing need for animal protein in the future.

**Abstract:**

We investigated the effect and interaction of lysolecithin (LPL) and nutrition level on growth performance, nutrient ileal digestibility, expression of growth-related genes and nutrient transporter genes in broilers. A total of 1280 one day old Ross 308 mixed sex chicks with an average body weight 42.23 ± 2.4 g were randomly allotted into 2 × 2 factorial arrangement (20 replicates per treatment and 16 chickens per replicate) with two types of diet (Normal nutrition treatments starter, grower and finisher diets with ME of 3000 kcal/kg, 3100 kcal/kg and 3200 kcal/kg, respectively, and CP level of 22%, 21%, and 20%, respectively; high nutrition treatments diets with 50 kcal/kg ME and 0.5% CP higher than normal nutrition treatment at each stage). Two levels of LPL supplementation (0 and 500 mg/kg) were also employed. From day 21 to day 35 and full stage of the experiment, the birds fed a high nutrition (HN) diet had a greater body weight gain (BWG) and lower feed conversion ratio (FCR) than those fed a normal nutrition (NN) diet (*p* < 0.05). Besides, lysolecithin increased BWG significantly (*p* < 0.05). The birds fed a diet with LPL revealed increasing fat digestibility compared to birds fed the basal diet (*p* < 0.05). LPL significantly increased the ileal digestibility of amino acids, including Ile, Thr, Phe, His, Arg, Tyr, Glu, Pro, Gly, Ala (*p* < 0.05). No interaction was found between LPL and nutrition level in BWG, FCR and nutrient digestibility. In HN diet, the genes expression of myogenic differentiation 1 (MYOD1), myogenin (MYOG), cluster of differentiation 36 (CD36), fatty acid-binding protein (FABP1), cationic amino acid transporter 1 (CAT1) and Y + L amino acid transporter 1 (y+, LAT1) were significantly elevated via LPL supplementation (*p* < 0.05). In NN diet, LPL significantly increased the genes expression of growth hormone (GH), insulin-like growth factor 1 (IGF1), MYOD1 and y+, LAT1 (*p* < 0.05). In conclusion, upregulating the nutrients transporter gene and growth-related gene expression of the host, independent of nutrition level changes, may be the action mechanism of lysolecithin on growth promotion in animals.

## 1. Introduction

LPL produced by phospholipase hydrolysis of soy lecithin has been widely used as a feed additive in the animal feed industry for over 20 years. It has been well recognized for improving animal growth performance and reducing feed costs [1]. Due to the removal of fatty acid by phospholipase, LPL has higher hydrophilic and oil-in-water properties [2]. Therefore, LPL has the capacity to improve the emulsification and digestion of fats and oils. Moreover, fats in chyme affect other feed components, which can interfere with utilization of other nutrients. Thus, the improved emulsification of fat could enhance the digestion of other nutrients [3]. In terms of absorption, LPL can alter phospholipid bilayers of enterocytes. The incorporation of LPL into cell membranes causes local deformation of bilayers, increasing fluidity and permeability of the membrane [4]. In addition, LPL can alter protein channel formation to increase ion exchanges [5]. Thus, these two factors can assist nutrients to pass the enterocytes membrane more efficiently, resulting in an improvement in nutrient absorption. Glucose, fatty acids and amino acids are transported to the small intestine via a transport system expressed within the intestinal cells. However, whether LPL can regulate nutrient transporters expression has not been reported.

Similar to mammals, growth and development in chickens are mainly regulated by the somatotropic axis, such as growth hormone (GH), growth hormone-releasing hormone (GHRH), insulin-like growth factors (IGF1 and IGF2), growth suppressor (SS), their associated carrier proteins and receptors, and other hormones [6]. GH can stimulate differentiation and proliferation of bone and muscle cells [7]. IGF1 is a peptide hormone that mediates many of the growth-promoting activities of GH in poultry [8]. Additionally, Myoblasts play a crucial role in skeletal muscle formation during broiler growth. Myogenic differentiation1 (MYOD1) and myogenin (MYOG) are important components of myogenic regulatory factors (MRFs) which dominate myoblast differentiation [9]. Saxena’s study showed that energy and protein significantly affect the expression of growth-related genes [10]. As we know, LPL consists of four main contents: lysophosphatidyl-choline (LPC), lysophosphatidyl-inositol (LPI), lysophosphatidyl-ethanolamine (LPE) and lysophosphatidic acid (LPA). Juntanapum found that LPC supplementation improved the laying hens’ feed efficiency via increasing fat digestibility and the uptake of amino acids or cholesterol to the enterocyte, upregulating the expression of some amino acids and cholesterol transporter genes [11]. Another study observed that supplementation of LPL elicits gene expression in the intestinal epithelium, leading to enhanced collagen deposition and villus length. On the contrary, purified LPC alone as a supplement does not mimic these responses [12]. Therefore, it might be a response of another lysolecithin or a combinatorial response of several lysolecithins. In terms of nutrient utilization, LPI has been documented to increase the expression of the lipogenic gene and stimulate the release of insulin [13,14]. Insulin signaling is a strong activator of mammalian target rapamycin (mTOR), which functions as a nutrient, energy and redox sensory, controls protein synthesis and regulates cellular metabolism, growth and proliferation [15,16]. Besides, LPI has been identified as endogenous ligand for G-protein coupled receptor 55 (GPR55) [17]. LPI binds to GPR55 which is coupled to activation of protein kinase B (Akt), signaling factors that are associated mTOR [18]. mTOR leads to the production of IGF-1 and IGF-2 [19]. Therefore, LPI, which might play an important role in animal growth, could be a new opportunity for LPL utilization.

To better understand the effect on feed supplementation of LPL on host we conducted this trial in the hope that it can provide some insight into improving growth performance and decreasing cost of animal feed in the husbandry industry.

## 2. Materials and Methods

The animal experimental protocol used in this study was approved by the Animal Care and Use Committee of Dankook University. The approval protocol number is DKU-20-1881.

### 2.1. Experimental Design and Animals

A total of 1280 one day old Ross 308 mixed sex chicks with an average body weight of 42.23 ± 2.4 g were randomly allotted into 2 × 2 factorial arrangement (20 replicates per treatment and 16 chicks per replicate) with 2 diet types (Normal nutrition treatments starter, grower and finisher diets with the ME of 3000 kcal/kg, 3100 kcal/kg and 3200 kcal/kg, respectively, and CP level of 22%, 21%, and 20%, respectively; high nutrition treatments diets with 50 kcal/kg ME and 0.5% CP higher than normal nutrition treatment in each stage.) and two levels of LPL supplementation (0 and 500 mg/kg). LPL is a commercial product (Power LPI^TM^, composed of lysolecithin derived from hydrolysis of soybean phospholipid by phospholipase with LPC ≥ 2.5%, LPI ≥ 1.2%, LPE ≥ 1.2%, LPA ≥ 0.5%.) which was provided by Kemin (China) Technologies Co., Ltd. Broiler chickens were fed pellet feed at starter (0 to 7 d), grower (7 to 21 d) and finisher (21 to 35 d) periods. All diets were formulated to meet or exceed the recommendation of CVB 2018 for broiler chickens (Table 1) [20]. All birds were reared at a temperature-controlled house and caged in a three-layer stainless steel battery cage of identical size (124 cm wide × 64 cm long × 40 cm high). During the whole trial period, birds were allowed ad libitum access to water and feed. Room temperature was set at 33 ± 1 °C for the first 3 days, and then gradually adjusted to 22 °C until the end of the experiment. The humidity was set a range of 45% to 55% and a lighting program offered a 23 h photoperiod (23 h light:1 h dark) according to the management guidelines of commercial broilers.

### 2.2. Growth Performance and Apparent Ileal Digestibility

The body weight (BW) and feed intake (FI) of broilers were recorded in each pen at the end of 0, 7, 21 and 35 d. The data were used to calculate body weight gain (BWG), FI, and feed conversion gain (FCR) in each and at whole stage. The mortality was recorded daily to adjust FCR. FCR was calculated by FI/BWG. 

From d 29 to d 35, 2 g/kg chromium oxide (Cr_2_O_3_) was mixed into broiler’s diets as an indigestible marker for the determination of the nutrient coefficient apparent ileal digestibility [21]. From d 33 to d 35, 6 broilers per pen (24 broilers per treatment) were euthanized by severing a jugular vein. The ileum was ligated and then separated from the gastrointestinal tract and, subsequently, the ileal digesta on the pen basis was immediately stored in sealed bags at −20 °C and freeze-dried, after which they were finely grounded to pass through a 1 mm screen and were then stored in a freezer at −20 °C until analysis [22]. Dietary dry matter (DM) was analyzed by AOAC 2012 [23]. Individual amino acid composition was measured using an Amino Acid Analyzer (Beckman 6300, Beckman Coulter Inc., Fullerton, CA, USA) after 24 h of 6 N-HCl hydrolysis at 110 °C. Performic acid was used before hydrolysis to oxidize Met and Cys to methionine sulfone and cysteic acid. Nitrogen was determined by a Kjectec 2300 Nitrogen Analyzer (Foss Tecator AB, Hoeganaes, Sweden). Chromium levels were determined via UV absorption spectrophotometry (Shimadzu, UV1201, Japan). The ileal nutrient digestibility was then calculated relative to the Cr_2_O_3_ concentration. The gross energy was determined by measuring the heat of combustion in the samples using a Parr 6100 oxygen bomb calorimeter (Parr instrument Co., Moline, IL, USA).

### 2.3. RNA Extraction and qRT-PCR

The RNA extraction and qRT-PCR analysis were performed according to the method of Park et al. [9]. The detailed operation steps were as follow. The breast muscle, liver and small intestine tissue were isolated and their total RNA were extracted separately by a TRIzol reagent (Invitrogen, Carlsbad, CA, USA). cDNAs were synthesized from total RNA (1 µg) using the Maxima First Strand cDNA Synthesis Kit (Life Technologies, Carlsbad, CA, USA). The target gene primer sequences are presented in Table 2. Quantitative real-time polymerase chain reactions were conducted according to the standard operating procedures and conducted using the 7500 Fast Real-Time PCR System (Applied Biosystems, Foster City, CA, USA). In the process, the system condition was maintained at 94 °C for 30 s for denaturation and decreased to 59–61 °C for 30 s for the annealing process, then increased to 72 °C for 30 s for DNA extension. A single peak melting curve from the amplicon was taken for expression analysis and glyceraldehyde-3-phosphate dehydrogenase (GAPDH) was utilized as an endogenous control for normalization purposes. The quantitative expression number was calculated based upon the 2ΔΔCt method [24].

### 2.4. Statistical Analysis

Growth performance and nutrient digestibility were analyzed as a completely randomized design, with a 2 × 2 factorial arrangement, with the cage as the experimental unit, using GLM procedure [25]. The results were tested for the main effects of diet type and LPL supplementation, as well as their interaction. The significance level was set at *p* < 0.05. The differences in qRT-PCR between treatments were separated using Duncan’s multiple range test. Besides, a Student’s t-test was employed to determine the statistical differences in qRT-PCR between the groups with LPL and without LPL in RNA analysis. Results were considered significant at * *p* < 0.05 and ** *p* < 0.01.

## 3. Results

### 3.1. Growth Performance and Ileal Digestibility

In the current study, the results of growth performance and nutrient digestibility are presented in Table 3 and Table 4. No significant difference in growth performance was found in the first two periods in each group. (*p* > 0.05). During d21 to d35 and the overall experiment, the birds fed high nutrition (HN) diet had a greater BWG and lower FCR than that fed normal nutrition (NN) diet (*p* < 0.05). Besides, LPL supplementation increased BWG significantly (*p* < 0.05). 

No statistical difference in nutrient digestibility was observed between the normal nutrition diet and high nutrition diet group (*p* > 0.05). The birds fed the diet with LPL supplementation revealed increasing fat digestibility comparing to birds fed the basal diet (*p* < 0.05). LPL supplementation significantly increased the digestibility of amino acids, including Ile, Thr, Phe, His, Arg, Tyr, Glu, Pro, Gly and Ala (*p* < 0.05). 

### 3.2. Expression of Growth-Related Genes

**Figure 1:**
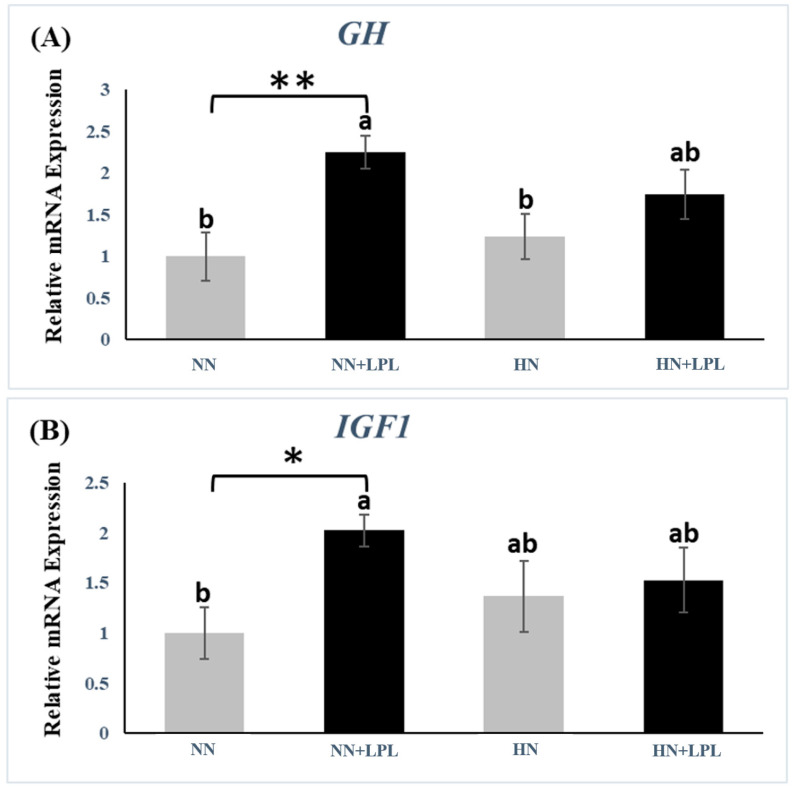
Quantitative genes expression of growth-related genes in liver following LPL supplementation. (**A**) The gene expression of GH. (**B**) The gene expression of IGF1. Significant differences between basal diet and diet with LPL supplementation groups are indicated by * *p* < 0.05 and ** *p* < 0.01. a,b Bars with no common letter differ significantly (*p*  <  0.05). Error bars indicate the standard error of the mean. IGF, insulin-like growth factor 1; GH, growth hormone. Abbreviation: NN, Normal nutrition diet; NN + LPL, NN + 500 g/t of LPL; HN, NN + 0.5% CP and 50 Kcal/kg; HN + LPL, HN + 500 g/t LPL.

**Figure 2:**
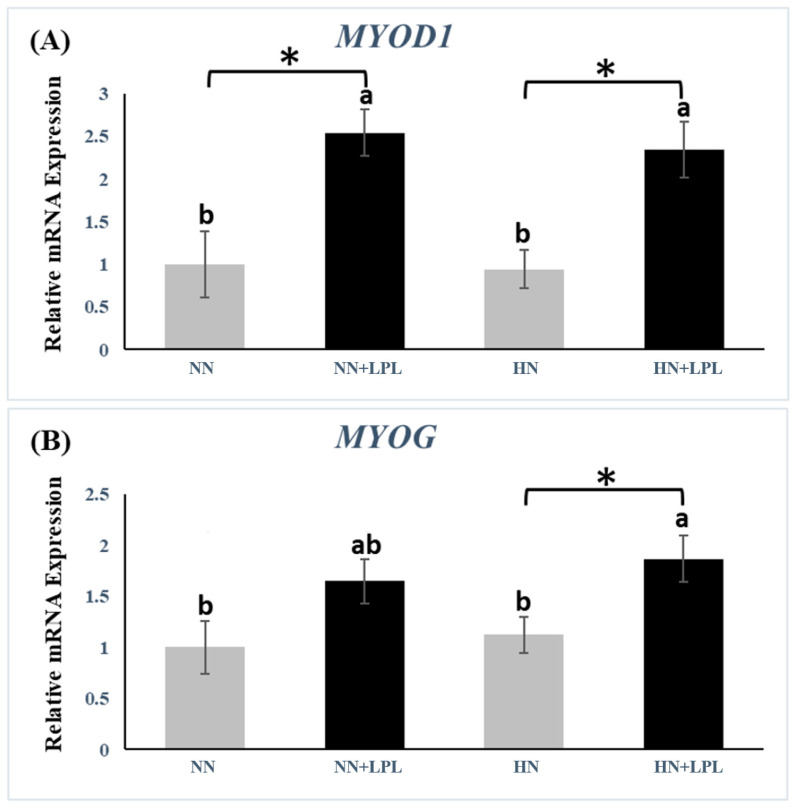
Quantitative genes expression of growth-related genes in muscle following LPL supplementation. (**A**) The gene expression of MYOD1. (**B**) The gene expression of MYOG. Significant differences between basal diet and diet with LPL supplementation groups are indicated by * *p* < 0.05. a,b Bars with no common letter differ significantly (*p*  <  0.05). Error bars indicate the standard error of the mean. MYOD1, myogenic differentiation 1; MYOG, myogenin. Abbreviation: NN, Normal nutrition diet; NN + LPL, NN + 500 g/t of LPL; HN, NN + 0.5% CP and 50 Kcal/kg; HN + LPL, HN + 500 g/t LPL.

To determine the effect of LPL supplementation on the expression of growth-related genes, the gene expression of insulin-like growth hormone (GH) and growth factor 1(IGF1) were examined in liver tissue, and the gene expression of myogenic differentiation1 (MYOD1) and myogenin (MYOG) were tested in muscle tissue (Figure 1 and Figure 2). The gene expression of GH (*p* < 0.01), IGF1 (P < 0.05) and MYOD1(*p* < 0.05) was increased in NN diet via LPL supplementation. In addition, the gene expression of MYOD1 and MYOG were elevated in HN diet via LPL supplementation (*p* < 0.05). However, there was no significant difference in growth-related genes between NN and HN diets (*p* > 0.05). 

### 3.3. Expression of Nutrient Transporter Genes

**Figure 3:**
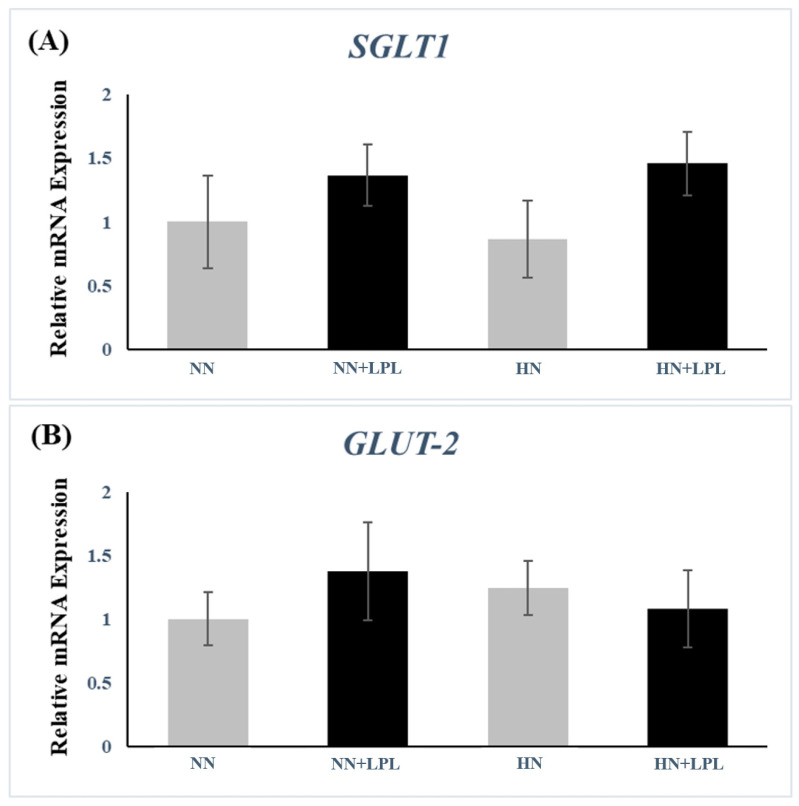
Quantitative gene expression of glucose transporter in small intestine following LPL supplementation. (**A**) The gene expression of SGLT1. (**B**) The gene expression of GLUT2. Error bars indicate the standard error of the mean. SGLT1, sodium-dependent glucose transporter 1; GLUT2, glucose transporter 2. Abbreviation: NN, Normal nutrition diet; NN + LPL, NN + 500 g/t of LPL; HN, NN + 0.5% CP and 50 Kcal/kg; HN + LPL, HN + 500 g/t LPL.

**Figure 4:**
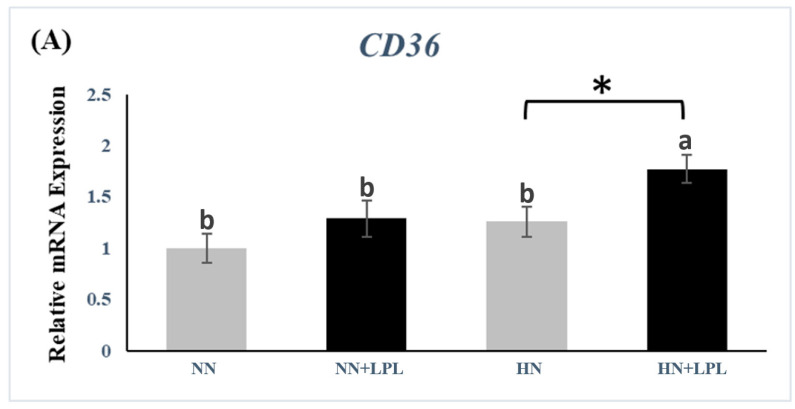

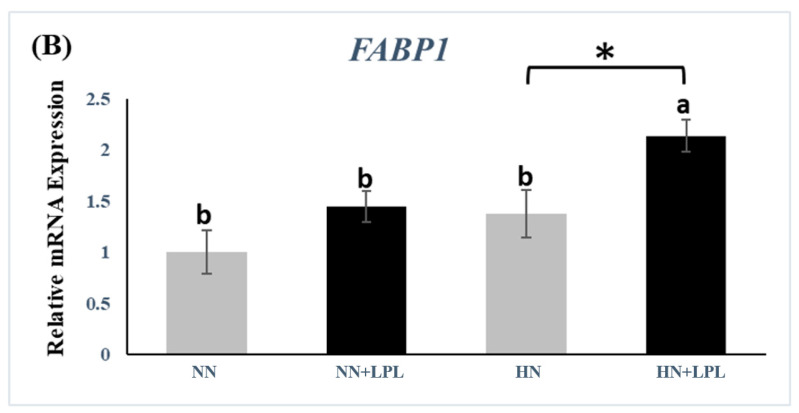
Quantitative gene expression of fat acids transporter in small intestine following LPL supplementation. (**A**) The gene expression of CD36. (**B**) The gene expression of FABP1. Significant differences between basal diet and diet with LPL supplementation groups are indicated by * *p* < 0.05. a,b Bars with no common letter differ significantly (*p*  <  0.05). Error bars indicate the standard error of the mean. CD 36, cluster of differentiation 36; FABP1, fatty acid-binding protein 1. Abbreviation: NN, Normal nutrition diet; NN + LPL, NN + 500 g/t of LPL; HN, NN + 0.5% CP and 50 Kcal/kg; HN + LPL, HN + 500 g/t LPL.

**Figure 5:**
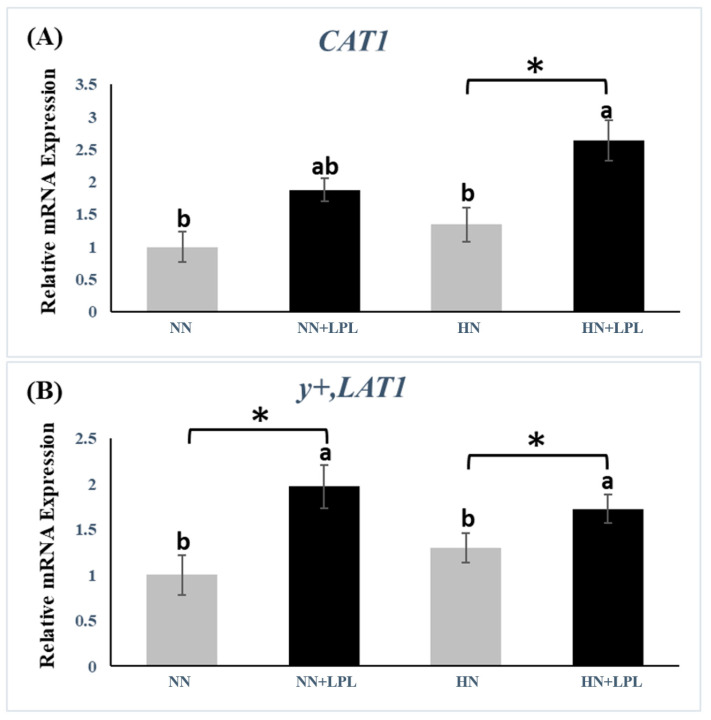
Quantitative gene expression of amino acids transporter in small intestine following LPL supplementation. (**A**) The gene expression of CAT1. (**B**) The gene expression of y+, LAT1. Significant differences between basal diet and diet with LPL supplementation groups are indicated by * *p* < 0.05. a,b Bars with no common letter differ significantly (*p*  <  0.05). Error bars indicate the standard error of the mean. CAT1, cationic amino acid transporter 1; y+, LAT1, Y + L amino acid transporter 1. Abbreviation: NN, Normal nutrition diet; NN + LPL, NN + 500 g/t of LPL; HN, NN + 0.5% CP and 50 Kcal/kg; HN + LPL, HN + 500 g/t LPL.

To evaluate the effect of LPL supplementation on the gene expression of nutrient transporter (glucose, fat acids and amino acids), the gene expression of sodium-dependent glucose transporter 1 (SGLT1), glucose transporter 2 (GLUT2), cluster of differentiation 36 (CD36), fatty acid-binding protein (FABP1), cationic amino acid transporter 1 (CAT1) and Y + L amino acid transporter 1 (y+, LAT1) were examined in small intestinal tissue (Figure 3, Figure 4 and Figure 5). Both nutrition level and LPL supplementation did not affect the gene expression of SGLT1 and GLUT2 significantly (*p* > 0.05). 

LPL supplementation increased the gene expression of CD36 and FABP1 in HN diet significantly (*p* < 0.05). Besides, the greater gene expression of CD36 and FABP1 was observed in HN + LPL group than in other treatments (*p* < 0.05). 

The gene expression of y+, LAT1 was increased via LPL supplementation (*p* < 0.05). However, no significant difference was observed on the gene expression of y+, LAT1 between NN and HN groups (*p* < 0.05). Besides, HN + LPL group revealed greater gene expression of CAT1 than other treatments (*p* < 0.05).

## 4. Discussion

### 4.1. Growth Performance and Ileal Digestibility of Animo Acids

Our previous study indicated that LPL supplementation improved FCR and total tract nutrient retention of energy in broiler chickens fed a low energy diet [26]. Similarly, Zhao et al. reported that birds fed LPL supplementation diet had a higher BWG, AME, DM digestibility and Lower FCR than those fed the diet without LPL supplementation [27]. Besides, a meta-analysis of 33 trials on LPL application in broilers concluded that, corrected FCR was not significantly affected in reformulated trials, suggesting that LPL supplementation at 125 and 250 g/t could recover average dietary energy reductions of 57.88 and 73.11 kcal/kg feed, respectively [28]. In agreement with these positive results, we found that LPL supplementation could benefit BWG. Although the effect of LPL addition on FCR is not significant, the data showed a decrease in absolute value. As referred to above, the functionality of LPL in facilitating fat emulsification has been generally accepted. Fat globules cannot be easily digested through enzymatic reaction and persist as indigestible residues within the intestinal tract. Besides, indigestible fat residues incorporate or cover other nutrient molecules, resulting in a negative impact of all nutrient digestion. Therefore, improving lipid emulsification benefits not only fat digestion but also other nutrients. 

Meanwhile, LPL can increase the fluidity and permeability of the cell membrane, which contributes not only to a decreasing deformation energy, but also the coupling between integral membrane proteins and their surrounding lipid bilayers will alter the hydrophobic interface to enter the protein channel [4,29]. These changes lead to a greater flux of several nutrients, boosting absorption of lipid and lipophilic nutrients. As soon as LPL is taken up by the enterocyte, it will be converted to phospholipids, important for chylomicrons, leading to sufficient lipid to contribute to growth [30]. In this study, LPL supplementation significantly increased the digestibility of amino acids, including Ile, Thr, Phe, His, Arg, Tyr, Glu, Pro, Gly and Ala. This is different from previous findings by Boontiam et al., who reported that no difference in total amino acid digestibility was detected via LPL supplementation [31]. As we know, LPL consists of four main contents: LPC, LPI, LPE, LPA. Various types, contents, and even the composition ratio of LPL might influence amino acids digestion and absorption in different ways. The main function of LPC is to promote oil emulsification and absorption; LPA not only promotes the migration and proliferation of intestinal epithelial cells, but also activates the Akt signaling pathway, thereby strengthening the mTOR signaling pathway. As mentioned above, LPI can activate the mTOR signaling pathway. According to these data, we speculate that LPI and LPA might be able to affect protein utilization. Current gene test data confirmed our hypothesis, which demonstrated that LPL supplementation raised gene expression of amino acid transporters. The differences in these studies may be due to the differences in the composition and origin of LPL. No interaction was found between LPL and nutrition level. This means that the changes in nutrition level in this paper cannot alter the effect of LPL on improving digestibility of amino acids and growth promotion in broilers. 

### 4.2. Growth-Related Genes 

In general, The GH/IGF-1 (insulin-like growth factor-1) axis plays the main role in animal growth. GH is secreted by the anterior pituitary gland and the primary effect is the activation of GH receptors and the secretion of IGF-1, mainly by the liver [32]. The effects of GH are mediated by the transmembrane GH receptors, which are expressed on the surface of most cells [33]. Therefore, GH plays an important role in growth via two independent mechanisms of action: one is to activate the cellular GH receptors and another is to induce IGF1 secretion by the liver. In this study, we found that LPL increased the gene expression of GH and IGF1. However, we could not find any direct evidence from previous publications. LPI supplementation has been reported to promote insulin release in a manner having the characteristics of physiologic exocytosis [14]. Besides, insulin seems to increase hepatic IGF1 expression [34]. Nam et al. confirmed a positive correlation between the serum total IGF-1 and insulin concentrations [35]. Thus, we hypothesize that LPI supplementation can increase IGF1 production via stimulating insulin release. In this study, there were two interesting findings. First, the increase of nutrition level (50 kcal/kg ME and 0.5% CP) did not influence the gene expression of GH and IGF1 in the liver. Some studies have reported that the plasma IGF1 concentration can be altered via nutritional status [36,37]. Saxena et al. reported that increasing energy (100 kcal/kg ME) and protein (1% CP) resulted in better growth performance of broiler chickens with corresponding upregulation of GH and IGF1 in the liver [10]. The inconsistency of IGF1 and GH expression may be due to the different nutrition gaps. Secondly, LPL can upregulate GH and IGF1 gene expression of birds fed NN diet but cannot affect that of birds fed HN diet. This might imply that reformulation (decreasing nutrition level) could be a more efficient method for LPL application in practice. 

MYOD1 is mainly required for myoblast proliferation, whereas MYOG is essential for terminal differentiation [38]. Current results indicated that LPL upregulated the MYOD1 gene expression when birds are fed NN and HN diet. However, LPL only significantly increased the MYOG in the HN group. It has been proven that differentiation and hypertrophy of myoblasts are regulated by the IGF-1 signaling pathway, which is critically mediated by the mTOR [39,40]. mTOR signaling pathway can integrate both intracellular and extracellular signals, and is a crucial central regulator of cell metabolism, growth, and proliferation. There may be the possibility that LPI could upregulate the IGF1/Akt signaling pathway via raising PtdIns-3-OH kinase (PI(3)K) production, resulting in an increased expression of mTOR. Akt/mTOR/S6K1 (protein S6 kinase-1) is a typical signaling pathway to control protein synthesis, which could induce changes in MYOD1 and MYOG gene expression [41]. Therefore, LPL might increase the expression of MYOD1 and MYOG gene expression via the IGF1/Akt/mTOR/S6K signaling pathway. However, we did not observe the increasing production of IGF1 while upregulating MYOD1 and MYOG gene expression in the HN group. Thus, there may be another possibility. As mentioned above, LPI is an endogenous ligand of GPR55 which leads several downstream signaling pathways. GPR55 can stimulate PLC activity, including Ca^2+^ release from the endoplasmic reticulum and activation of various protein kinase C (PKC) isoforms [42]. PKC might control mTOR expression via PKC/Akt signaling pathway, resulting in the regulation of protein synthesis. Therefore, LPL might increase MYOD1 and MYOG gene expression via the GPR55/PKC/Akt/mTOR/S6K signaling pathway. Generally, protein and amino acids play an important role in growth signals to regulate protein metabolism via modulating the translation of initiation and elongation factors [43]. Neutral aliphatic AA, including Met and the branched-chain AA (BCAA), has been reported to stimulate S6K1, a downstream target of the mTOR signaling pathway, which initiates protein synthesis [44]. However, our data indicated that the diet nutrition level did not influence the expression of MYOD1 and MYOG. It might that 0.5% CP difference is not enough to impact signaling, or that protein level has nothing to do with that signaling. In addition, the HN + LPL groups had higher MYOG gene expression relative to the other treatments. This suggests that there is an interaction between nutrient level and LPL, which can help to promote MYOG gene expression. 

### 4.3. Nutrient Transporter Genes

Nutrient digestibility is closely associated with the expression of intestinal nutrient transporters. CD36, which is sited in the brush border membrane of enterocytes in the duodenum and jejunum, is important for chylomicron production and acute fatty acid uptake [45]. Khonyoung et al. demonstrated that LPL supplementation could enhance lipid absorption via greater CD36 reaction [46]. Juntanapum et al. reported that LPC supplementation could increase fat digestibility via upregulation of Niemann-Pick C1-like 1 (NPC1), one kind of sterol transporter [11]. Consistent with those results and current digestibility data, LPL increased the gene expression transporters of fatty acids (CD36 and FABP1). However, no reference on the effect of LPL supplementation on intestinal FABP1 was found. Interestingly, we only found the significant effect of LPL in the HN diet. It seems that the combination of dietary fat content and LPL contribute to the CD36 and FABP1 production in the small intestine. 

Both CAT-1 and y+, LAT1 are the major system y+ transporter in most cells (system y+, with its ability to recognize cationic amino acids (lysine and arginine) [47]. The paper of Juntanapum et al. revealed that the expression of CAT1 gene was elevated by LPC supplementation in lying hens’ feed [11]. In agreement with his results, we found LPL increased the expression of CAT1 and y+, LAT1. On the one hand, the upregulation of nutrient transporters might be one kind of adaptive response to an improvement in nutrient digestibility, especially fat and protein. On the other hand, m-TOR participates in normal adipose tissue growth, and regulates fat cell and whole-body organ size and systemic glucose and lipid metabolism, which might also influence the intestinal fatty acids transporters (CD36 and FABP1) in terms of lipid absorption [48]. Thus, LPL can increase CD36 and FABP1 production via GPR55/PKC/Akt/mTOR or IGF1/Akt/mTOR signaling pathway. As with fatty acid transporter gene expression, this possibility might exist in amino acid absorption. 

Interestingly, the difference in nutrition level did not change CAT1 and y+LAT1 production. However, Osmanyan et al. documented that the CAT1 expression could be influenced by the change of dietary protein (1.5% CP) in broilers [49]. Similarly, Garcia et al. found the different dietary protein change (9.0% CP) could affect the CAT1 production in growing pigs [50]. The various results could be due to the difference in the protein gap, as 0.5% protein difference may not be enough to change the amino acids transporter production. Other than that, we did not observe any significant effect of LPL on glucose transporter (SGLT1 and GLUT2). Therefore, the effect of LPL on broiler growth performance enhancement is achieved largely through regulation of fat and amino acids transporter gene expression. 

## 5. Conclusions

In conclusion, supplementation of LPL in diet can improve growth performance (BWG) and nutrient ileal digestibility (especially amino acids) of broilers via enhancing the expression fatty acid transporter (CD 36, FABP1) genes, amino acid transporters (CAT1, y+, LAT1) genes and growth-related genes (IGF1, GH, MYOD1 and MYOG), independently of nutrition level. This might be the key mechanism of action of LPL in promoting the growth of farm animals.

## Figures and Tables

**Table 1:** Basal diet composition (as-fed basis).

Ingredient, %	Starter ^1^		Grower ^1^		Finisher ^1^	
	Normal	High	Normal	High	Normal	High
Corn	59.03	56.25	58.05	57.27	61.50	58.72
Soymeal meal (46%)	25.24	27.15	24.18	24.44	18.92	20.54
Hydrolyzed Render Meal (50%)	3.00	3.00	4.00	4.00	5.00	5.00
Corn gluten meal (60%)	5.00	5.00	5.00	5.00	5.00	5.00
DDGS (Maize)	3.00	3.00	3.00	3.00	3.00	3.00
Soy oil	1.65	2.54	3.00	3.52	3.99	5.18
Limestone	0.22	0.24	0.12	0.13	0.07	0.08
Calcium hydro-phosphate	1.15	1.13	1.06	1.04	1.02	1.00
Salt	0.31	0.31	0.31	0.31	0.31	0.31
Methionine (99%, DL-Form)	0.43	0.44	0.40	0.40	0.33	0.33
Lysine -HCl (98.5%)	0.50	0.48	0.43	0.44	0.44	0.42
Threonine (98.5%)	0.17	0.16	0.15	0.15	0.12	0.12
Choline (60%)	0.10	0.10	0.10	0.10	0.10	0.10
Vitamin premix ^2^	0.10	0.10	0.10	0.10	0.10	0.10
Mineral premix ^3^	0.10	0.10	0.10	0.10	0.10	0.10
Total	100	100	100	100	100	100
Calculated composition, %						
Crude protein	22	22.5	21	21.5	20	20.5
Metabolism energy (kcal/kg)	3000	3050	3100	3150	3200	3250
Calcium	0.95	0.95	0.9	0.9	0.85	0.85
Available phosphorus	0.40	0.40	0.38	0.38	0.36	0.36
SID-Lys	1.23	1.25	1.15	1.17	1.05	1.07
SID-Met + SID-Cys	0.96	0.98	0.90	0.91	0.82	0.83
SID-Thr	0.65	0.66	0.61	0.62	0.56	0.57

^1^ Starter diets, provided during weeks 0 to 1; grower diets, provided during weeks 1 to 3; finisher diets, provided during weeks 3 to 5. ^2^ Provided per kg of diet: 15,000 IU of vitamin A, 3750 IU of vitamin D3, 37.5 mg of vitamin E, 2.55 mg of vitamin K3, 3 mg of thiamin, 7.5 mg of riboflavin, 4.5 mg of vitamin B6, 24 μg of vitamin B12, 51 mg of niacin, 1.5 mg of folic acid, 0.2 mg of biotin and 13.5 mg of pantothenic acid. ^3^ Provided per kg of diet: 37.5 mg Zn (as ZnSO_4_), 37.5 mg of Mn (MnO_2_), 37.5 mg of Fe (as FeSO_4_•7H_2_O), 3.75 mg of Cu (as CuSO_4_•5H_2_O), 0.83 mg of I (as KI), and 0.23 mg of Se (as Na_2_SeO_3_•5H_2_O).

**Table 2:** Primers sequences used in the real-time RT-PCR analysis.

NO.	Gene Symbol	Accession No	Primer (5′ to 3′)	
			Forward	Reverse
1	SGLT1	NM_001293240	TTAGAGAGGTTGGAGGGTATGA	GAATCTGCTCGAGGCGTATAG
2	GLUT2	NM_207178	AGAGGAAACTGTGACCCGATGA	AACGAAGAGGAAGATGGCGA
3	CD36	NM_204192	GAAGGTCTGAGCCCAAATGA	AGGTGTCACAAGGAGGTTTAC
4	FABP1	NM_001293240	ACTGGCTCCAAAGAATGACCAATG	TGTCTCCGTTGAGTTCGGTCAC
5	CAT1	NM_001145490	CTTGATCGCTGCCTTGGCTT	CCGTAATGAAGGCCCACAGC
6	y+ LAT1	XM_001231336	GCCAACTAGCCAGGCGGTTA	TATCCTGCACCCGTGTTCCC
7	IGF1	NM_001004384	TGCTGCTTCCAGAGTTGTGACC	TGGCATATCAGTGTGGCGCT
8	GH	NM_204359	TACGGCCTGCTGTCCTGCTT	TGTTTTTGGTGACGGGGAGG
9	MYOD1	NM_204214	GGCCGCCGATGACTTCTATG	TGCTCCTCCTCGTGTGGGTT
10	MYOG	NM_204184	AGCGATGACCAGGCAGAGGA	CCAGCTCAGTTTTGGACCCG

**Table 3:** Effect of LPL supplementation in diet with different nutrition content on growth performance in broilers ^1^.

Items	Normal Nutrition		High Nutrition		SEM ^2^	*p*-Value ^3^		
	NN	NN + LPL	HN	HN + LPL		Feed Effect	LPL Effect	Interaction
d 1 to 7				.				
BWG, g	125	128	128	130	2	0.3127	0.1617	0.7677
FI, g	147	152	153	153	3	0.091	0.2916	0.4229
FCR	1.176	1.188	1.195	1.177	0.028	0.8886	0.8925	0.6745
d 7 to 21								
BWG, g	657	660	657	668	7	0.605	0.3309	0.5947
FI, g	1008	1017	1036	1031	9	0.1580	0.8706	0.3651
FCR	1.534	1.541	1.577	1.544	0.018	0.1812	0.4659	0.2156
d 21 to 35								
BWG, g	945	965	974	988	16	0.0430	0.008	0.8686
FI, g	1749	1763	1726	1742	14	0.2035	0.3878	0.9607
FCR	1.851	1.826	1.772	1.763	0.021	0.0070	0.4807	0.8516
Overall								
BWG, g	1727	1753	1760	1786	16	0.0445	0.0022	0.9837
FI, g	2904	2931	2915	2925	18	0.8963	0.3501	0.6658
FCR	1.682	1.673	1.658	1.6385	0.01	0.0124	0.2079	0.6299

^1^ Abbreviation: NN, Normal nutrition diet; NN + LPL, NN + 500 g/t of LPL; HN, NN + 0.5% CP and 50 Kcal/kg; HN + LPL, HN + 500 g/t LPL. BWG, body weight gain; FI, feed intake; FCR, feed conversion ratio. ^2^ Standard error of means. ^3^ *p* < 0.05 was considered statistically significant, whereas *p* < 0.10 was considered a tendency.

**Table 4:** Effect of LPL supplementation in diet with different nutrition content on ileal digestibility in broilers ^1^.

Items, %	Normal Nutrition		High Nutrition		SEM ^2^	*p*-Value ^3^		
	NN	NN + LPL	HN	HN + LPL		Feed Effect	LPL Effect	Interaction
day 35								
Dry matter	70.76	71.26	72.11	71.99	0.75	0.394	0.879	0.800
Nitrogen	68.32	71.68	69.91	72.19	1.23	0.345	0.854	0.717
Energy	67.37	70.91	72.06	71.77	0.71	0.314	0.855	0.692
Fat	86.71	88.10	85.77	88.08	0.63	0.550	0.026	0.567
Val	88.42	91.14	88.84	90.01	0.86	0.744	0.078	0.474
Met	89.91	91.38	90.64	92.15	0.8	0.334	0.058	0.978
Ile	86.49	90.14	86.00	89.84	0.71	0.689	0.004	0.926
Leu	86.64	89.50	86.42	89.76	1.63	0.993	0.092	0.893
Thr	88.01	90.38	87.27	91.56	0.89	0.879	0.025	0.506
Phe	81.51	84.58	81.33	84.82	0.78	0.980	0.005	0.855
His	86.35	87.94	84.88	88.64	0.59	0.640	0.002	0.197
Lys	87.90	90.42	86.70	90.60	0.93	0.667	0.275	0.565
Arg	86.94	89.71	86.42	89.22	0.61	0.695	0.034	0.988
Trp	78.15	80.00	76.21	82.80	1.13	0.795	0.105	0.160
Tyr	88.31	91.42	87.54	90.97	0.7	0.619	0.010	0.897
Ser	86.10	88.01	86.03	88.15	0.95	0.979	0.938	0.938
Glu	85.08	87.60	84.55	87.87	0.6	0.905	0.010	0.716
Pro	86.20	88.93	85.46	88.50	0.86	0.528	0.003	0.870
Gly	86.43	88.83	86.00	88.61	0.78	0.771	0.031	0.926
Ala	88.82	91.79	88.27	92.52	0.56	0.940	0.005	0.608
Cys	86.26	88.99	83.95	89.96	1.08	0.594	0.176	0.197
Asp	88.85	91.83	88.15	91.17	1.02	0.597	0.245	0.989

^1^ Abbreviation: NN, Normal nutrition diet; NN + LPL, NN + 500 g/t of LPL; HN, NN + 0.5% CP and 50 Kcal/kg; HN + LPL, HN + 500 g/t LPL. ^2^ Standard error of means. ^3^ *p* < 0.05 was considered statistically significant, whereas *p* < 0.10 was considered a tendency.

## Data Availability

Data supporting reported results can be requested from the corresponding author via email.

## References

[1] Haetinger V.S., Dalmoro Y.K., Godoy G.L. (2021). Optimizing cost, growth performance, and nutrient absorption with a bio-emulsifier based on lysophospholipids for broiler chickens. Poult. Sci.

[2] Joshi A., Paratkar S.G., Thorat B.N. (2006). Modification of lecithin by physical, chemical and enzymatic methods. Eur. J. Lipid Sci..

[3] Honda K., Kamisoyama H., Isshiki Y., Hasegawa S. (2009). Effects of dietary fat levels on nutrient digestibility at different sites of chicken intestines. JPSA.

[4] Lundbæk J.A., Andersen O.S. (1994). Lysophospholipids modulate channel function by altering the mechanical properties of lipid bilayers. J. Gen. Physiol..

[5] Maingret F., Patel A.J., Lesage F., Lazdunski M., Honoré E. (2000). Lysophospholipids open the two-pore domain mechanogated K(+) channels TREK-1 and TRAAK. J. Biol. Chem..

[6] Renaville R., Hammadi M., Portetelle D. (2002). Role of the somatotropic axis in the mammalian metabolism. Domest. Anim. Endocrinol..

[7] Kühn E.R., Vleurick L., Edery M., Decuypere E., Darras V.M. (2002). Internalization of the chicken growth hormone receptor complex and its effect on biological functions. Comp. Biochem. Physiol. Part B Biochem. Mol. Biol..

[8] Anh N.T.L., Kunhareang S., Duangjinda M. (2015). Association of Chicken Growth Hormones and Insulin-like Growth Factor Gene Polymorphisms with Growth Performance and Carcass Traits in Thai Broilers. Asian-Australas. J. Anim. Sci..

[9] Park J.H., Lee S.I., Kim I.H. (2020). The effect of protease on growth performance, nutrient digestibility, and expression of growth-related genes and amino acid transporters in broilers. J. Anim. Sci. Technol..

[10] Saxena R., Saxena V.K., Tripathi V., Mir A., Dev K., Begum J., Agarwal R., Goel A. (2020). Dynamics of gene expression of hormones involved in the growth of broiler chickens in response to the dietary protein and energy changes. Gen. Comp. Endocrinol..

[11] Juntanapum W., Bunchasak C., Poeikhampha T., Rakangthong C. (2020). Poungpong, K; Effects of supplementation of lysophosphatidylcholine (LPC) to lying hens on production performance, fat digestibility, blood lipid profile, and gene expression related to nutrients transport in small intestine. J. Anim. Feed Sci..

[12] Brautigan D.L., Li R., Kubicka E., Turner S.D., Garcia J.S., Weintraut M.L., Wong E.A. (2017). Lysolecithin as feed additive enhances collagen expression and villus length in the jejunum of broiler chickens. Poult. Sci..

[13] Moreno-Navarrete J.M., Catalan V., Whyte L., Diaz-Arteaga A., Vazquez-Martinez R., Rotellar F. (2012). The L-alpha-lysophosphatidylinositol/GPR55 system and its potential role in human obesity. Diabetes.

[14] Metz S.A. (1986). Lysophosphatidylinositol, but not lysophosphatidic acid, stimulates insulin release. A possible role for phospholipase A2 but notde novosynthesis of lysophospholipid in pancreatic islet function. Biochem. Biophys. Res. Commun..

[15] Menon D., Salloum D., Bernfeld E., Gorodetsky E., Akselrod A., Frias M.A., Sudderth J., Chen P.H., DeBerardinis R., Foster D.A. (2017). Lipid sensing by mTOR complexes via de novo synthesis of phosphatidic acid. J. Biol. Chem..

[16] Yoon M.S. (2017). The Role of Mammalian Target of Rapamycin (mTOR) in Insulin Signaling. Nutrients.

[17] Oka S., Nakajima K., Yamashita A., Kishimoto S., Sugiura T. (2007). Identification of GPR55 as a lysophosphatidylinositol receptor. Biochem. Biophys. Res. Commun..

[18] Ross R.A. (2011). L-α-Lysophosphatidylinositol meets GPR55: A deadly relationship. Trends Pharmacol. Sci.

[19] Kimball S.R., Jefferson L. (2006). New functions for amino acids: Effects on gene transcription and translation. Am. J. Clin. Ntr..

[20] CVB (2018). CVB Table Booklet Feeding of Poultry. Voluntary Feed Intake in Pigs Feeding Standards, Feeding Advices and Nutritional Values of Feed Ingredients for Poultry.

[21] Sales J., Janssens G.P.J. (2003). The use of markers to determine energy metabolizability and nutrient digestibility in avian species. World’s Poult. Sci. J..

[22] Mountzouris K.C., Tsitrsikos P., Palamidi I., Arvaniti A., Mohnl M., Schatzmayr G., Fegero K. (2010). Effects of probiotic inclusion levels in broiler nutrition on growth performance, nutrient digestibility, plasma immunoglobulins, and cecal microflora composition. Poult. Sci..

[23] AOAC (2012). Official Methods of Analysis.

[24] Livak K.J., Schmittgen T.D. (2001). Analysis of relative gene expression data using real-time quantitative PCR and the 2−ΔΔCT method. Methods.

[25] SAS Institute (1998). SAS User’s Guide: Statistics.

[26] Park J.H., Nguyen D.H., Kim I.H. (2018). Effects of Exogenous Lysolecithin Emulsifier Supplementation on the Growth Performance, Nutrient Digestibility, and Blood Lipid Profiles of Broiler Chickens. J. Poult. Sci..

[27] Zhao P.Y., Kim I.H. (2017). Effect of diets with different energy and lysophospholipids levels on performance, nutrient metabolism, and body composition in broilers. Poult. Sci..

[28] Wealleans A.L., Jansen M., Benedetto M.D. (2019). The addition of lysolecithin to broiler diets improves growth performance across fat levels and sources: A meta-analysis of 33 trials. Br. Poult. Sci..

[29] Lundbæk J.A., Collingwood S.A., Ingólfsson H.I., Kapoor R., Andersen O.S. (2010). Lipid bilayer regulation of membrane protein function: Gramicidin channels as molecular force probes. J. R. Soc. Interface.

[30] Boontiam W., Jung B., Kim Y.Y. (2017). Effects of lysophospholipid supplementation to lower nutrient diets on growth performance, intestinal morphology, and blood metabolites in broiler chickens. Poult. Sci..

[31] Boontiam W., Hyun Y.K., Jung B., Kim Y.Y. (2019). Effects of lysophospholipid supplementation to reduced energy, crude protein, and amino acid diets on growth performance, nutrient digestibility, and blood profiles in broiler chickens. Poult. Sci..

[32] Giustina A., Veldhuis J.D. (1998). Pathophysiology of the neuroregulation of growth hormone secretion in experimental animals and the human. Endocr. Rev..

[33] Frago L.M., Paneda C., Dickson S.L., Hewson A.K., Argente J., Chowen J.A. (2002). Growth hormone (gh) and gh-releasing peptide-6 increase brain insulin-like growth factor-i expression and activate intracellular signaling pathways involved in neuroprotection. Endocrinology.

[34] Froesch E.R., Guler H.P., Schmid C., Zapf J. (1990). Therapeutic potential of insulin-like growth factor 1. Trends. Endocrinol. Metab..

[35] Nam S.Y., Lee E.J., Kim K.R., Cha B.S., Song Y.D., Lim S.K., Lee H.C., Huh K.B. (1997). Effect of obesity on total and free insulin-like growth factor (IGF)-1, and their relationship to IGF-binding protein (BP)-1, IGFBP-2, IGFBP-3, insulin, and growth hormone. Int. J. Obes..

[36] Fekete S.G., Brown D.L. (2007). Veterinary aspects and perspectives of nutrigenomics: A critical review. Acta Vet. Hung..

[37] Lu F.Z., Chen J., Wang X.X., Liu H.L. (2009). Investigation of the Insulin-like growth factor system in breast muscle during embryonic and postnatal development in langshan and arbor acres chickens subjected to different feeding regimens. Asian-Aust. J. Anim. Sci..

[38] Megeney L.A., Rudnicki M.A. (1995). Determination versus differentiation and the MyoD family of transcription factors. Biochem. Cell Biol..

[39] Devol D.L., Rotwein P., Sadow J.L., Novakofski J., Bechtel P.J. (1990). Activation of insulin-like growth factor gene expression during work-induced skeletal muscle growth. Am. J. Physiol. Endocrinol. Metab..

[40] Coleman M.E., DeMayo F., Yin K.C., Lee H.M., Geske R., Montgomery C., Schwartz R.J. (1995). Myogenic vector expression of insulin-like growth factor I stimulates muscle cell differentiation and myofiber hypertrophy in transgenic mice. J. Biol. Chem..

[41] Wang Y.J., Ma J.D., Qiu W.L., Zhang J.W., Feng S.Y., Zhou X.K., Wang X., Jin L., Long K., Liu L.Y. (2018). Guanidinoacetic Acid Regulates Myogenic Differentiation and Muscle Growth Through miR-133a-3p and miR-1a-3p Co-mediated Akt/mTOR/S6K Signaling Pathway. Int. J. Mol. Sci..

[42] Zeng Y., Irvine R., Hiley C. (2015). Biased signalling might be the answer to the inconsistent pharmacology of GPR55. FASEB J..

[43] Metayer S., Seiliez I., Collin A., Duchêne S., Mercier Y., Geraert P.A., Tesseraud S. (2008). Mechanisms through which sulfur amino acids control protein metabolism and oxidative status. J. Nutr. Biochem..

[44] Zeitz J.O., Mohrmann S., Käding S.C., Devlikamov M., Niewalda I., Whelan R., Helmbrecht A., Eder K. (2018). Effects of methionine on muscle protein synthesis and degradation pathways in broilers. J. Anim. Physiol. Anim. Nutr..

[45] Drover V.A., Ajmal M., Nassir F., Davidson N.O., Nauli A.M., Sahoo D., Tso P., Abumrad N.A. (2005). CD36 deficiency impairs intestinal lipid secretion and clearance of chylomicrons from the blood. J. Clin. Investig..

[46] Khonyoung D., Yamauchi K., Suzuki K. (2015). Influence of dietary fat sources and lysolecithin on growth performance, visceral organ size, and histological intestinal alteration in broiler chickens. Livest. Sci..

[47] Hatzoglou M., Fernandez J., Yaman I., Closs E. (2004). Regulation of cationic amino acid transport: The story of the CAT-1 transporter. Annu. Rev. Nutr..

[48] Mao Z., Zhang W.Z. (2018). Role of mTOR in Glucose and Lipid Metabolism. Int. J. Mol. Sci..

[49] Osmanyan A.K., Shahab G.H., Reza M., Fisinin V.I., Arkhipova A.L., Glazko T.T., Kovalchuk S.N., Kosovsky G.Y. (2017). Intestinal amino acid and peptide transporters in broiler are modulated by dietary amino acids and protein. Amino Acids.

[50] Garcia V.H., Adriana M.T., Araiza-Piña B.A., Htoo J.K., Cervantes-Ramírez M. (2012). Effects of dietary protein and amino acid levels on the expression of selected cationic amino acid transporters and serum amino acid concentration in growing pigs. Arch. Anim. Nutr..

